# Fiber-optic control and thermometry of single-cell thermosensation logic

**DOI:** 10.1038/srep15737

**Published:** 2015-11-13

**Authors:** I.V. Fedotov, N.A. Safronov, Yu.G. Ermakova, M.E. Matlashov, D.A. Sidorov-Biryukov, A.B. Fedotov, V.V. Belousov, A.M. Zheltikov

**Affiliations:** 1 Physics Department, International Laser Center, M.V. Lomonosov Moscow State University, Moscow 119992, Russia; 2 Department of Physics and Astronomy, Texas A&M University, College Station TX 77843, USA; 3 Russian Quantum Center, ul. Novaya 100, Skolkovo, Moscow Region, 143025 Russia; 4 M.M. Shemyakin and Yu.A. Ovchinnikov Institute of Bioorganic Chemistry, Russian Academy of Sciences, Moscow 117997, Russia; 5 Kurchatov Institute National Research Center, Moscow 123182, Russia

## Abstract

Thermal activation of transient receptor potential (TRP) cation channels is one of the most striking examples of temperature-controlled processes in cell biology. As the evidence indicating the fundamental role of such processes in thermosensation builds at a fast pace, adequately accurate tools that would allow heat receptor logic behind thermosensation to be examined on a single-cell level are in great demand. Here, we demonstrate a specifically designed fiber-optic probe that enables thermal activation with simultaneous online thermometry of individual cells expressing genetically encoded TRP channels. This probe integrates a fiber-optic tract for the delivery of laser light with a two-wire microwave transmission line. A diamond microcrystal fixed on the fiber tip is heated by laser radiation transmitted through the fiber, providing a local heating of a cell culture, enabling a well-controlled TRP-assisted thermal activation of cells. Online local temperature measurements are performed by using the temperature-dependent frequency shift of optically detected magnetic resonance, induced by coupling the microwave field, delivered by the microwave transmission line, to nitrogen—vacancy centers in the diamond microcrystal. Activation of TRP channels is verified by using genetically encoded fluorescence indicators, visualizing an increase in the calcium flow through activated TRP channels.

Technologies allowing the electrical activity of specific cells in a living organism to be controlled with a high resolution both in space and time offer new, unprecedented opportunities for the functional analysis of complex biological systems. Optogenetic methods^1^,^2^,^3^,^4^,^5^,^6^,^7^ employ genetically encoded light-sensitive ion channels for a spatially precise selective modulation of specific cells within complex distributed networks of neurons, thus offering unique tools for studying the mechanisms whereby the dynamics of these networks controls cognitive responses, memory, learning, and behavior. As a promising alternative to optogenetic strategies, thermogenetics uses thermosensitive ion channels^8^,^9^,^10^,^11^,^12^,^13^,^14^,^15^,^16^,^17^,^18^ to drive the cell activity by temperature variations. However, since temperature changes affect many physiological processes in a living organism, cell activation by temperature variations requires special precautions in order to avoid heating that would be incompatible with the general physiology of the organism and to make sure that temperature variations are small enough to prevent increased background activity of cells, such as a spontaneous firing of neurons. These difficulties limit neuroscience applications of thermogenetics primarily to experiments with fruit flies^12^,^18^, despite the availability of a broad variety of thermosensitive members within the vast family of TRP channels, covering broad ranges of working temperatures and activation thresholds, possessing an exceptional thermal sensitivity, and allowing neurons expressing these channels to be switched from silent to robustly active mode by a slight change in temperature.

In this work, we demonstrate a technique that addresses these issues by allowing the temperature of individual cells be controlled in a highly accurate and well-localized fashion. Our approach is based in a specifically designed fiber probe that can induce a localized, precisely controlled heating of individual cells expressing genetically encoded heat-sensitive TRPA channels in a cell culture. A diamond microcrystal attached to the tip of the fiber^19^ and heated by laser radiation transmitted through the fiber provides a local heating of the cell culture, enabling a well-controlled TRP-assisted thermal activation of cells. Moreover, this fiber probe can simultaneously measure the temperature of a cell through a temperature-dependent frequency shift of optically detected magnetic resonance, which is induced by coupling the microwave field, delivered by the microwave transmission line^19^, to the spin of nitrogen–vacancy (NV) centers in diamond on the tip of the fiber probe.

Experiments on thermogenetic control and thermometry of single cells were performed (Fig. 1) on a culture of Human Embryonic Kidney 293 (HEK-293) cells grown in a Petri dish. The HEK-293 cells were transfected with vectors expressing G-GECO 1.2 calcium indicator^20^ and rattlesnake TRPA1 channels, known to be responsible for remote thermosensation by rattlesnake *Crotalus atrox*^14^. A Petri dish with the cell culture was placed on a translation stage on a high-precision adjustable microscope table. Cell imaging was performed using a 10 × microscope objective and a CCD camera. The cells were irradiated with a continuous-wave 473-nm diode-laser output, which provided optical excitation of G-GECO 1.2.

The fiber probe, positioned in the cell culture using a homebuilt high-precision mechanical manipulator, serves to deliver continuous-wave 532-nm laser radiation, giving rise, through a laser-induced heating of the diamond, to a spherical gradient of temperature in the cell culture, which falls off with the distance r from the diamond microcrystal (Fig. 1). The same fiber probe is used to measure the temperature at the chosen site within the cell culture. To this end, the microwave field, delivered through the two-wire microwave transmission line integrated with the fiber (Fig. 2), is applied to couple the spin sublevels of ground-state NV centers in diamond, polarized by 532-nm laser radiation transmitted through the optical tract of the fiber probe (Fig. 1). This laser radiation transfers population from the ^3^*A* ground state to the ^3^*E* excited state. The photoluminescence (PL) emitted as a result of this process features a zero-phonon line at approximately 637 nm, observed as a well-resolved peak on a broad phonon-sideband line. This PL signal is collected by the same optical fiber^19^. The optical tract of the fiber then serves to transmit this signal to the detection system, which consists of a silicon photodiode, a low-noise preamplifier, and a lock-in amplifier (Fig. 1).

In the absence of external magnetic fields, the *m*_s_ = 0 and *m*_s_ = ±1 sublevels of the ground-state triplet of NV centers are split by Ω_s_ ≈ 2.87 GHz. The 532-nm optical field spin-polarizes NV centers, accumulating them in the *m*_s_ = 0 state through spin-selective decay paths^21^,^22^,^23^. Since the population from the *m*_s_ = ±1 excited state can be transferred to the *m*_s_ = 0 level through a metastable singlet state, which does not fluoresce within the 630–800-nm band, the PL yield of NV centers in the *m*_s_ = ± 1 state is lower than the PL yield of *m*_s_ = 0 NV centers. The intensity of the PL signal, *I*_PL_, therefore decreases when a microwave field delivered by the transmission line integrated into our fiber probe is tuned to the zero-field splitting frequency Ω_s_, transferring population from the *m*_s_ = 0 state to the *m*_s_ = ± 1 sublevels. This effect is observed as a well resolved feature in the PL intensity *I*_PL_ measured as a function of the microwave frequency Ω. Even in the absence of external magnetic fields, a local strain removes the degeneracy of this resonance, giving rise to two well-resolved features in the optically detected magnetic resonance (ODMR) spectra *I*_PL_(Ω) (the inset in Fig. 3a). As the temperature of diamond increases, this profile of the zero-external-magnetic-field resonance is shifted, as shown in the earlier work^24^, toward lower microwave frequencies, enabling temperature measurements with a high spatial resolution. For the highest sensitivity and highest speed of local temperature measurements in a cell culture, frequency-modulated microwave spin excitation in NV centers was combined with properly optimized differential lock-in detection^25^,^26^. In a recent work^27^, a system consisting of an optical fiber, NV-diamond sensor, and a microwave transmission line has been used to demonstrate a thermogenetic activation of single cells by microwave radiation. In experiments presented here, the power of the microwave field was at least three orders of magnitude lower to avoid any effects induced by the microwave field.

In a calibration experiment, the fiber probe was placed inside a thermostat with a physiological solution at a precisely controlled temperature along with a thermocouple, providing an accuracy of temperature measurements higher than 0.1 °C. Figure 3a displays the magnetic resonance zero-field splitting frequency Ω_s_ measured as a function of the temperature inside the thermostat according to thermocouple readings. As can be seen from this plot, a linear function with a slope *d*Ω_s_/*dT* ≈ −75 ± 2 kHz/K provides an ideal fit for this dependence within the entire temperature range of interest, viz., from 34 °C to 49 °C, offering a convenient calibration for temperature measurements using our fiber-optic probe with NV diamond.

In another test experiment, the temperature of NV diamond on the tip of the optical fiber was measured, through the temperature-dependent shift of ODMR spectra, as described above, as a function of the power of 532-nm laser radiation in a thermostat with physiological solution and with air. The results of these measurements are presented in Fig. 3b. Each temperature reading was taken following sufficiently long time interval of heating with a fixed continuous-wave laser power (longer than 1 min) to make sure that the heat transfer in our system had reached the steady-state regime, where the incoming heat flux, provided by laser radiation, is equal to the outgoing heat flux due to a heat sink through the bottom of the Petri dish, the optical fiber, and the interface between the cell culture and the air. To verify that, following the initial transient phase right after a change in the heating laser power, heat transfer in our system occurs in the steady-state regime, we performed measurements of ODMR spectra within a 1-min time interval, making sure these spectra exhibit no changes that would be indicative of nonstationary heat transfer due to an unbalanced incoming heat flux. Since the thermal conductivity of air, *k*_a_ ≈ 0.026  W/(m K), is much lower than the thermal conductivity of the solution, *k*_s_ ≈ 0.6 W/(m K), for each given power of 532-nm laser radiation, the temperature of the diamond in air is noticeably higher than its temperature in solution, exactly as one would expect in a situation when laser radiation first heats the diamond particle, which, in its turn, transfers heat to the surrounding medium. The temperature of diamond linearly grows with the laser power (Fig. 3b), which is also consistent with the standard heat-conduction model.

In a separate experiment, we verified the validity of the steady-state solution of the heat-conduction equation for the temperature of the cell culture, *T*(*r*) = (*R*_d_/*r*)(*T*_d_ − *T*_0_) +*T*_*0*_, where *R*_d_ is the radius of the diamond microscrystal, *r* ≥ *R*_d_ is the distance from the diamond microcrystal, *T*_d_ is the temperature of the diamond microcrystal, which is measured directly in experiments, and *T*_0_ is the temperature at a distant boundary (at the infinity in mathematical terms), set equal to the room temperature for our system (*T*_0_ ≈ 21 °C). In this experiment, we directly measured the temperature of the cell culture as a function of *r* using a second fiber-optic NV-diamond probe with a diameter of 30 μm. The average power of 532-nm laser radiation used in this fiber-probe was kept below 1 mW to avoid additional heating of the culture. The results of these measurements (circles in Fig. 3c), do not deviate from the 1/*r* steady-state solution to the heat-conduction equation (the dashed line in Fig. 3c) by more than a few percent.

In our experiment with a culture of HEK-293 cell expressing the rattlesnake TRPA1 channel and G-GECO 1.2 calcium indicator, we first focus on the thermal response of a group of nine closely spaced cells within the field of view of the microscope (Fig. 3d). To find the temperatures at the locations of individual cells in this group, we define the temperature of the diamond microcrystal from the temperature-dependent shift of the magnetic resonance zero-splitting frequency of NV centers, read out through the fiber probe as described above, and apply the equation for the spherical temperature gradient *T*(*r*) to calculate *T*(*r*_*i*_) for each *r*_*i*_. To make sure that the steady-state temperature is measured, temperature readings in these measurements are taken at least 100 s after a change in the laser power *P*_0_. We then plot the calcium indicator fluorescence intensity *I*_f_ as a function of the local temperature *T*(*r*_*i*_) for each cell of this group (Fig. 3e). It is straightforward to see from the typical *I*_f_(*T*) curve in Fig. 3e that, right below the cell activation threshold, the fluorescence intensity *I*_f_ starts to rapidly grow, taking off from its background level (shown by the dashed line in Fig. 3e). This rising section of the *I*_f_(*T*) curve is accurately approximated by a linear function (dash–dotted line in Fig. 3e). Following Gracheva *et al.*^14^, we define the activation threshold, *T*_a_, of a cell expressing rattlesnake TRPA channels as an intercept of the linear approximation of *I*_f_(*T*) extrapolated beyond the rising section of *I*_f_(*T*) and the fluorescence background line (Fig. 3e). As can also be seen from Fig. 3e, the fluorescence signal continues to increase with growing *P*_0_, due to a gradual increase in the number of opening channels on cell membranes, until all the TRPA1 channels are activated, at which point the fluorescence signal starts to saturate. Such a behavior of *I*_f_(*T*) makes the temperature *T*_m_, corresponding to the median point in the rising section of *I*_f_(*T*), a meaningful parameter of cell thermosensation. The histogram in Fig. 3f shows the activation thresholds *T*_a_ and *T*_m_ defined for all the nine cells under study. Averaging over these results yields the mean values of *T*_a_ and *T*_m_ for the studied group of cells along with their standard deviations: *T*_a_ ≈ 27.3 ± 0.6 °С and *T*_m_ ≈ 28.0 ± 0.5 °С.

It is important to note that, when applied to the thermal activation of cells, the standard deviation, which has been estimated as *σ* ≈ 0.6 °С for the studied group of cells, is more than a mere measure of instrumental errors. Since the opening temperatures vary from channel to channel, the standard deviation, apart from instrumental errors, provides a measure for the intrinsic uncertainty of cell activation threshold definition and quantifies the extent to which thermal activation in an ensemble of cells can be controlled.

To demonstrate a thermal activation of individual cells, we focus on three HEK-293 cells in the same group of nine cells in the field of view of the microscope in our experiment (Fig. 3d). These three cells under study (Fig. 4) are located at distances *r*_1_ ≈ 373 μm, *r*_2_ ≈ 401 μm, and *r*_3_ ≈ 421 μm from the NV diamond microcrystal on the tip of the fiber probe. In Fig. 4, the temperatures of individual cells *T*(*r*_*i*_) are shown as functions of the laser power *P*_0_ delivered to the diamond microcrystal on the fiber tip. As can be seen from these plots, the temperature of each cell grows monotonically in response to an increase in *P*_0_, with the temperatures of cells lying closer to the diamond microcrystals being always higher than the temperatures of cells with larger *r*_*i*_. This monotonic growth in cell temperatures is verified by the fluorescence of G-GECO 1.2 indicator, which becomes progressively brighter as *P*_0_ increases.

Since the first cell is closer to the heat source, its temperature, *T*_1_ = *T*(*r*_1_), is always slightly higher than the temperatures *T*_2_ = *T*(*r*_2_) and *T*_3_ = *T*(*r*_3_) of the other two cells. At low levels of *P*_0_, the intensity of G-GECO 1.2 fluorescence from all the cells remains low (image 1 in Fig. 4). At the level of laser radiation powers such that the temperature of the first cell reaches *T*_m_, but the temperatures of the second and third cells are still lower than *T*_m_ (*P*_0_ ≈ 59 mW in Fig. 4), the first cell is observed as a bright green spot in the image of the cell culture, due to intense G-GECO 1.2 fluorescence, indicating the flow of Ca^2+^ through the cell membrane, while the second and third cells are still dark (image 2 in Fig. 4). With *P*_0_ ≈ 68 mW, the temperature of the second cell reaches *T*_m_, and this cell is also clearly visible as a bright green spot in the image of the cell culture (image 3 in Fig. 4). At this point, of the three cells under study, only the third cell remains dark, as its temperature is lower than *T*_m_. Finally, with *P*_0_ ≈ 77 mW, the temperatures of all three cells is higher than *T*_m_. At this level of laser radiation powers, all the cells are observed as bright green spots due to intense fluorescence of G-GECO 1.2 (image 4 in Fig. 4).

To summarize, thermal activation and online thermometry of individual cells have been demonstrated using a fiber-optic probe integrated with an NV-diamond quantum sensor. A diamond microcrystal on the fiber tip is heated by laser radiation transmitted through the fiber, providing a local heating of the cell culture, enabling a well-controlled TRPA1-assisted thermal activation of cells. Online local temperature measurements have been performed using the temperature-dependent frequency shift of optically detected magnetic resonance, induced by coupling the microwave field to NV centers in diamond on the tip of the fiber probe. Individual activation of TRPA1-channel-expressing cells has been independently verified in our experiments by using genetically encoded fluorescence indicators, visualizing an increase in the calcium flow through activated TRPA channels.

## Methods

### Fiber probe design

A fiber probe used in our experiments integrates^19^,^26^ an optical fiber, an NV-diamond microcrystal, and a two-wire microwave transmission line. For the NV-diamond sensor, we use high-pressure high-temperature diamond microcrystals enriched with NV centers (Fig. 2b), as described in Refs 19,26, up to an NV center density of 10^16^–10^17^ cm^–3^. With a help of mechanical manipulator, a diamond microcrystal 30–250 μm in diameter is attached, under an optical microscope, to the tip of an optical fiber with a core diameter of 200 μm and a numerical aperture NA ≈ 0.2 and fixed to the fiber tip with ethyl cyanoacrylate glue.

**Figure 1:**
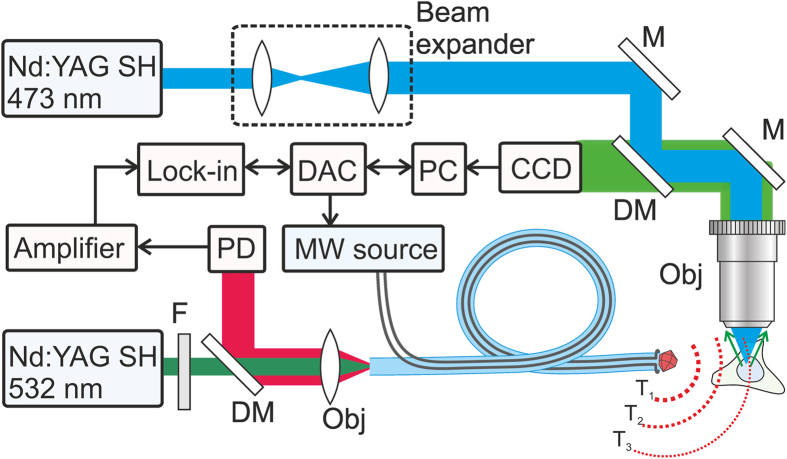
Thermogenetics using a fiber-optic probe, serving as a local heater and thermometer: Nd: YAG SH, second-harmonic output of a continuous-wave Nd: YAG laser; M, mirrors; DM, dichroic mirrors; PMT, photomultiplier tube; DAC, digital-to-analog converter; PD, photodetector; MW source, microwave source; F, filter; GM, galvanoscanned mirror; Obj, objective. The dotted lines show the surfaces of equal temperature visualizing the temperature gradient induced by the diamond microparticle on the tip of the fiber probe.

**Figure 2:**
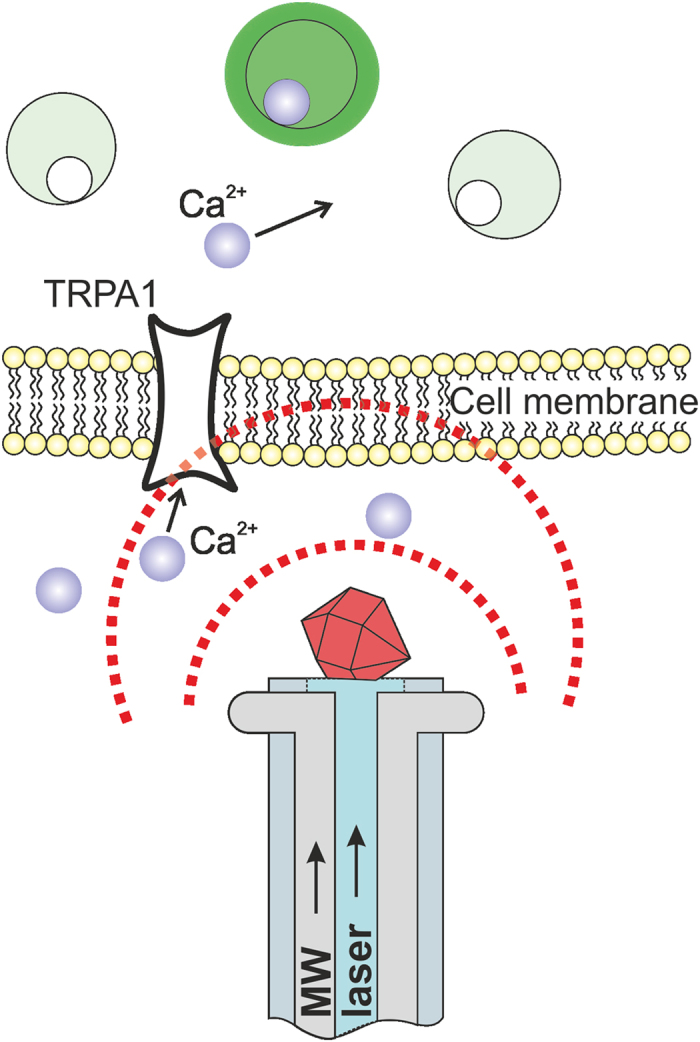
Local heating and thermometry of cells expressing genetically encoded thermosensitive TRP channels and Ca^2+^ indicator. As the heat delivered to the cell by a laser-heated diamond on the tip of the fiber is increased to a level where the temperature of the cell becomes higher than the TRP channel activation threshold, the flow of Ca^2+^ ions through TRP channels increases, making the G-GECO 1.2 Ca^2+^ indicator flash green.

The electron spin of NV centers is manipulated through the electron spin resonance induced by a microwave field, which is delivered to the diamond microcrystal with NV centers along a two-wire transmission line, which consists of a pair of copper wires 50 μm in diameter each, running along the optical fiber (Figs 1 and 2). A loop that short-circuits this transmission line near the fiber tip with a diamond microcrystal (Figs 1 and 2) provides a microwave field distribution with a maximum at the location of the diamond microcrystal.

### Cell culture and transfection

HEK-293 cells (ATCC) were seeded into 35 mm glass bottom dishes (MatTek) and cultured in DMEM with 10% FCS (PAA Laboratories) at 37 °C in a 5% CO_2_ atmosphere, as described in detail in ref. 28. After 24 hours cells were transfected by a mixture of 1 ng DNA (or 0.65 ng DNA of each vector for co-transfection) and 3 μl (6 μl for co-transfection) X-treme GENE 9 transfection reagent per one dish. After 8–10 hours cell medium was replaced by fresh medium. Some 36–48 hours after transfection, HEK-293 cells were incubated for 2 hours in MEM without bicarbonate supplemented with 20 mM of HEPES-NaOH pH 7.4 at 37 °C.

## Additional Information

**How to cite this article**: Fedotov, I.V. *et al.* Fiber-optic control and thermometry of single-cell thermosensation logic. *Sci. Rep.* **5**, 15737; doi: 10.1038/srep15737 (2015).

## Figures and Tables

**Figure 3:**
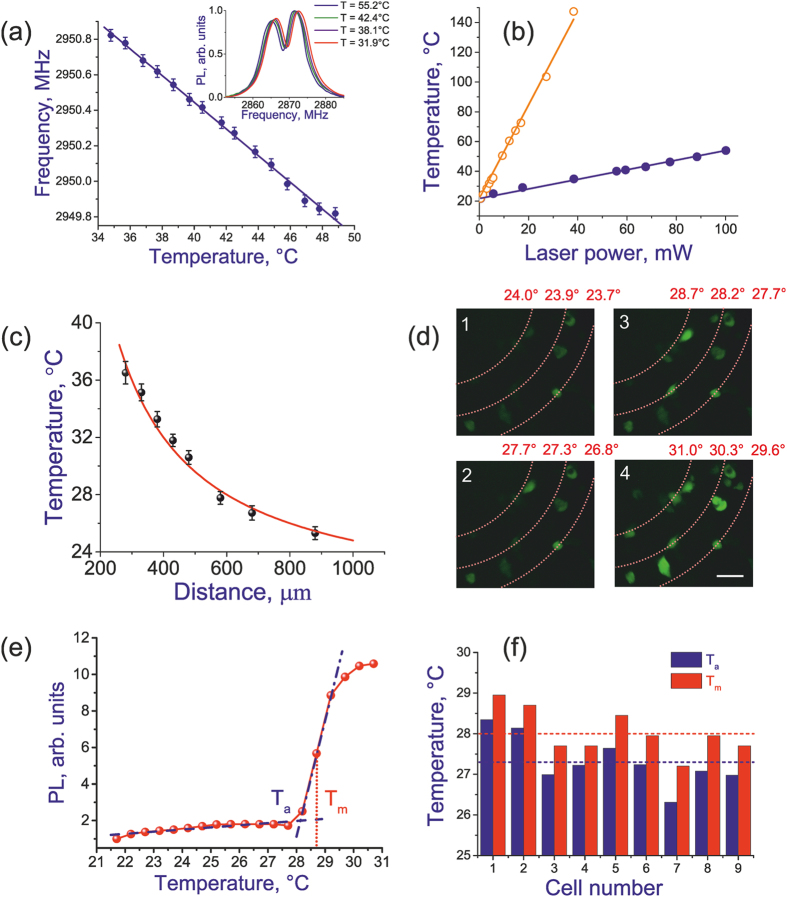
(**a**) The zero-field magnetic-resonance frequency as a function of the temperature measured by a thermocouple in a thermostat. The inset shows the intensity of photoluminescence from NV centers in a diamond microcrystal attached to the fiber tip measured as a function of the frequency of the microwave field delivered to the fiber tip through the microwave transmission line for different laser powers heating the diamond to temperatures ranging from about 31.9 °C to 55.2 °C, as specified in the plot. (**b**) The temperature of NV diamond on the tip of the optical fiber measured through the temperature-dependent shift of ODMR spectra as a function of the power of 532-nm laser radiation in a thermostat with physiological solution (filled circles) and with air (open circles). (**c**) The temperature of the cell culture measured as a function of the distance from the laser-heated NV-diamond microparticle with the use of a second fiber-optic NV-diamond sensor: (circles) experimental results and (solid line) the steady-state solution to the heat-conduction equation with R_d_ = 150 μm, T_d_ = 52 °C, and T_0_ = 21 °C. (**d**) The images of the cell culture taken with *P*_0_ ≈ 18 mW (*1*), 56 mW (*2*), 68 mW (*3*), and 88 mW (*4*). The scale bar is 50 μm. The fiber-coupled diamond microcrystal is located 325 μm above the plane of the images. The red concentric dotted lines show the lines of equal temperature. The temperatures inferred from NV-diamond-sensor measurements are indicated above the images. (**e**) The G-GECO 1.2 fluorescence intensity as a function of the temperature for the cell located at r = 371 μm. The background fluorescence level is shown by the dashed line. The dash—dotted line shows a linear approximation of the rising segment of the *I*_f_(*T*) dependence with its extrapolation beyond this segment. The intercept of these two lines is used as a definition of the cell activation threshold *T*_a_. (**f**) Activation thresholds *T*_a_ (blue) and *T*_m_ (red) defined for the nine cells under study. The mean values of these thresholds are shown by dashed lines.

**Figure 4:**
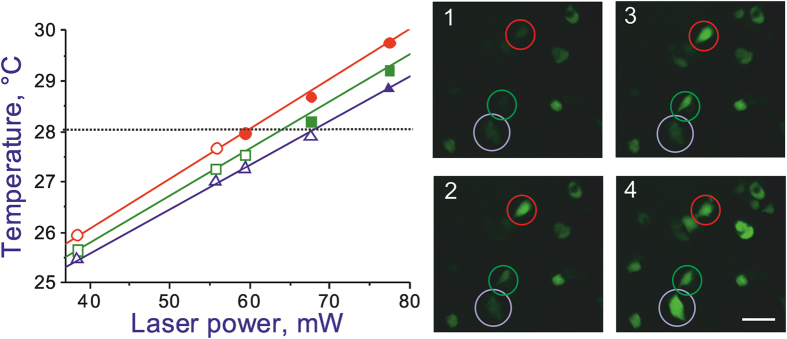
The temperatures of individual cells in the cell culture inferred from NV-diamond-sensor measurements as functions of the laser power delivered to the diamond microcrystal on the fiber tip. The median activation temperature *T*_m_ is shown by the dotted line. Also shown are the images of the cell culture at *P*_0_ = 38 mW (1), 59 mW (2), 68 mW (3), and 77 mW (4). The cells under study are circled. The scale bar is 50 μm.
